# The association of Social Anxiety Disorder, Alcohol Use Disorder and reproduction: Results from four nationally representative samples of adults in the USA

**DOI:** 10.1371/journal.pone.0188436

**Published:** 2017-11-21

**Authors:** Beyon Miloyan, Adam Bulley, Ben Brilot, Thomas Suddendorf

**Affiliations:** 1 Department of Mental Health, Bloomberg School of Public Health, Johns Hopkins University, Baltimore, Maryland, United States of America; 2 Department of Psychology, Faculty of Health, Federation University, Ballarat, Victoria, Australia; 3 School of Psychology, The University of Queensland, Brisbane, Queensland, Australia; 4 School of Biological Sciences, Plymouth University, Plymouth, United Kingdom; Technion Israel Institute of Technology, ISRAEL

## Abstract

Social Anxiety Disorder (SAD) and Alcohol Use Disorder (AUD) are highly prevalent and frequently co-occur. The results of population studies suggest that SAD tends to precede AUD, and the results of laboratory studies suggest that alcohol use facilitates social behaviors in socially anxious individuals. Therefore, we posited that, in a modern context, a tendency to consume alcohol may be positively selected for among socially anxious individuals by its effect on the likelihood of finding a partner and reproducing. We tested the hypothesis that a higher proportion of individuals with a lifetime diagnosis of SAD and AUD reproduce (i.e., have at least one child) relative to individuals with SAD absent AUD in an individual participant meta-analysis based on over 65,000 adults derived from four nationally representative cross-sectional samples. We then cross-validated these findings against the results of a 10-year follow up of one of these surveys. Lifetime history of SAD was not associated with reproduction whereas lifetime history of AUD was positively associated with reproduction. There was no statistically detectable difference in the proportion of individuals with a lifetime history of SAD with or without AUD who reproduced. There was considerable heterogeneity in all of the analyses involving SAD, suggesting that there are likely to be other pertinent variables relating to SAD and reproduction that should be delineated.

## Introduction

Population-based studies in the USA suggest that approximately 5 to 12% of adults have met criteria for Social Anxiety Disorder (SAD) in their lifetime [1,2]. Nearly half of those individuals meeting diagnostic criteria for a lifetime history of SAD also meet diagnostic criteria for a lifetime history of AUD [1,3]. SAD has been estimated to precede AUD in up to 80% of comorbid cases, and baseline SAD is associated with up to four times higher odds of developing AUD at follow-up [3,4]. Why might socially anxious individuals be prone to excessive alcohol use?

Social anxiety is thought to have evolved due to selective pressures on the mismanagement of social fitness threats (e.g., to one’s reputation) that can result in reduced cooperation and diminished access to mates or resources [5–8], and is thought to be unlikely to be a product of modern society [9,10]. Clinical social anxiety (i.e. SAD), in which dispositional responses to perceived social threats are triggered with high frequency in the course of daily life, can be considered maladaptive insofar as these responses engender the persistent avoidance of social situations due to a strong fear of embarrassment or humiliation. Such avoidance can cause significant distress because individuals with SAD crave social interactions [11], and when avoidance is not possible they tend to endure these situations with severe discomfort. Some degree of social anxiety can generally be beneficial as a means of managing potential social threats, for instance by means of appropriate regulation of social behavior in adhering to hierarchy (for evolutionary perspectives on social anxiety, see [8,12–16]). However, excessive social anxiety, as captured by a diagnosis of SAD, may interfere with daily activities and with fitness relevant behaviors, such as finding a partner.

Nesse and Berridge have suggested that psychoactive drug use in a modern context serves to artificially signal fitness benefits by acting on conserved emotion regulation circuitry that evolved in the absence of such stimuli [17]. From this perspective, by signaling the absence of social threats, alcohol consumption would be expected to down-regulate the typical anxiety response among socially anxious individuals, thereby facilitating social behaviors, with potentially positive implications for finding a reproductive partner [18]. Indeed, alcohol use is associated with reduced subjective anxiety, reduced amygdala activity, as well as less pronounced attentional and memory biases toward social threats among those with SAD [19–23]. Thus, socially anxious individuals may come to drink alcohol as a means of avoidance coping [24], and to facilitate more effective navigation of social situations [25,26].

Against this background, we sought to examine the current fitness implications of social anxiety (indexed by a diagnosis of SAD) with versus without excessive alcohol use (indexed by a diagnosis of AUD). We used four national samples of American adults, one of which was a 10-year longitudinal survey, to test the hypothesis that a lifetime history of SAD and AUD would be associated with higher odds of having reproduced (having at least one child) relative to individuals with a lifetime history of SAD without AUD.

## Materials and methods

### Samples

The National Epidemiological Survey on Alcohol and Related Conditions (NESARC) was undertaken in 2001–2002 by the U.S. Bureau of the Census and sponsored by the National Institute of Alcohol and Alcoholism (NIAAA) [27]. The NESARC was conducted in a national sample of 43,093 civilian, non-institutionalized adults (aged 18–98 years old) who were sampled from all 50 U.S. states and the District of Columbia. African-Americans, Hispanics, and young adults were purposively oversampled. The response rate was 81%. The subsequent surveys used in the present study were undertaken by the University of Michigan’s Survey Research Center [28]. The National Comorbidity Survey (NCS) was undertaken in 1990–1992 in a national sample of 8,098 civilian, non-institutionalized adults (aged 15–54 years old) who were sampled from the United States [29]. Of this initial sample, 5,001 participants were followed up 10 years later in Wave 2 (NCS-2) [30]. The National Comorbidity Survey Replication (NCS-R) was undertaken in 2001–2003 in a new nationally representative sample of 9,282 adults (aged 18–99 years old) who were sampled from the United States [31,32]. The National Latino and Asian American Study (NLAAS) was undertaken in 2002–2003 in a nationally representative sample of 4,649 Asian and Hispanic Americans (aged 18–97 years old) residing in the US [33]. Face-to-face interviews were conducted by trained lay interviewers in all of the surveys (see previous references for more information). See Table 1 for sociodemographic characteristics of the samples.

**Table 1:** Sociodemographic characteristics of the samples.

	*Raw frequencies (and weighted proportions)*			
	NESARC (N = 43,093)	NCS (N = 8,098)	NCS-R (N = 9,282)	NLAAS (N = 4,649)
Age	46 ± 18	33 ± 11	44 ± 18	39 ± 15
Sex				
Male	-	-	-	-
Female	24,439 (57%)	4,263 (51%)	5,143 (52%)	2,524 (50%)
Education				
Bachelor’s degree or higher	9,941 (25%)	1,813 (19%)	2,389 (24%)	1,238 (19%)
Some college	12,559 (30%)	2,132 (22%)	2,726 (28%)	1,096 (22%)
Completed high school	12,412 (29%)	2,679 (37%)	2,796 (32%)	1,005 (23%)
Less than high school	7,773 (16%)	1,474 (22%)	1,371 (16%)	1,310 (37%)
Lifetime Partner Status				
Married/cohabiting	21,958 (52%)	4,410 (63%)	5,322 (56%)	3,069 (66%)
Widowed/divorced/separated	11,048 (26%)	1,253 (10%)	2,017 (20%)	661 (13%)
Never married	9,679 (22%)	2,435 (27%)	1,943 (24%)	919 (22%)
Reproduction*				
No offspring	-	-	-	-
One or more	31,114 (74%)	3,372 (62%)	6,496 (69%)	3,341 (73%)
Lifetime Social Anxiety Disorder				
Absent	-	-	-	-
Present	2,018 (5%)	1,059 (13%)	1,143 (12%)	310 (7%)
Lifetime Alcohol Use Disorder				
Absent	-	-	-	-
Present	11,825 (28%)	1,921 (23%)	1,034 (12%)	299 (9%)
Lifetime SAD and AUD				
Absent	-	-	-	-
Present	924 (3%)	367 (4%)	282 (3%)	61 (2%)

*Reproduction was assessed in a representative subsample of the NCS, as part of an extended demographic interview

### Measures

Sociodemographic variables included age, sex and education (did not complete high school; completed high school or its equivalent; some college; bachelor’s degree or higher). Lifetime social anxiety disorder (SAD) and alcohol use disorder (AUD) diagnoses were based on the Alcohol Use Disorder and Associated Disabilities Interview Schedule (AUDADIS) and the Comprehensive International Diagnostic Interview (CIDI) diagnostic algorithms based on DSM-IV criteria, with test-retest reliability estimates ranging from fair to very good (kappa: 0.46–0.70) and good to excellent (kappa: 0.64–0.78), respectively [34,35]. Additional psychopathological variables included lifetime diagnosis of anxiety (Panic Disorder, Generalized Anxiety Disorder, and Specific Phobias) and mood disorders (Major Depression and Dysthymia). All of the datasets included a variable that captured the number of offspring that a respondent reported having. Given that approximately 80% of the respondents who had reproduced in each of the samples had three or fewer children, number of offspring was converted to a dichotomous variable.

### Statistical analyses

The NCS-R and NLAAS analyses were based on the full sample sizes of 9,282 and 4,649, respectively. In the NESARC analysis, four hundred and eight participants who did not provide data about offspring were excluded, resulting in a sample size of 42,685. The NCS was conducted in two parts: 8,098 respondents were administered a diagnostic interview that gathered data on basic sociodemographic characteristics and psychiatric diagnoses, and a subset of 5,877 of those respondents were administered an extended interview. Due to this design feature, the analysis on reproduction was based on the subsample of 5,877 participants.

First, a binary logistic regression model adjusting for sociodemographic characteristics, including age, sex and educational attainment, and psychiatric characteristics, including lifetime history of anxiety (excluding SAD) and mood disorders was developed to assess the association between SAD and AUD as predictors, and reproduction as outcome across all of the datasets. These psychiatric controls were included because alternative hypotheses have posited that alcohol use patterns may be accounted for by distress due to psychiatric conditions more generally (e.g., the Self Medication Hypothesis) [36]. The analytic plan was developed in the NESARC, before obtaining access to the other datasets. In the first analysis, SAD and AUD were entered individually into the model separately in order to assess the relationship of each disorder with the outcomes of the study. Then, in a second analysis, these two variables were replaced with an interaction variable comparing respondents with a lifetime history of SAD with and without a lifetime history of AUD to controls on reproduction. Based on this second analysis, the interaction of the odds ratios (ORs) obtained for those with SAD with and without AUD for each outcome was calculated by taking the natural log of the ORs of SAD with and without AUD [37]. The difference score of the two estimates was then exponentiated to reconvert the effect estimate into a Relative Odds Ratio (ROR). These analyses were repeated using the NCS, NCS-R and NLAAS, and the estimates were pooled using random-effects meta-analysis [38] which assumes a distribution of effect estimates.

The average age of onset estimates of SAD were 12 in the NCS-R, 13 in the NLAAS, 15 in the NCS, and 16 in the NESARC, and the average age of onset estimates of AUD were 20 in the NCS, and 22 in the NCS-R, NLAAS and NESARC. Therefore, in all of our datasets, as in previous studies [39,40], the average age of onset of SAD preceded that of AUD. However, due to the use of cross-sectional data, we were unable to assure that the required sequence of events was fully accounted for (e.g., due to recall error). We therefore performed an additional analysis using the 10-year follow-up of the National Comorbidity Surveys, to cross-validate the pooled results of the cross-sectional studies with longitudinal data. In the longitudinal analysis, the joint SAD and AUD group consisted of participants who reported a lifetime diagnosis of SAD prior to the past year and a past-year diagnosis of AUD at Wave 1. Reproduction was measured at Wave 2, only counting individuals whose first child was at most 10 years of age, to ensure that reproduction took place at least at the Wave 1 interview. As above, the model adjusted for age, sex, education and lifetime mood and anxiety disorders (excluding SAD). All analyses were survey-weighted, in order to adjust for the complex survey designs, including oversampling, non-response and attrition, and performed in Stata/SE 12.1 [41].

## Results

### Social Anxiety Disorder (SAD)

Binary logistic regression models were used to assess the association between lifetime history of SAD as a predictor and reproduction as outcome, adjusting for age, sex, education, and lifetime history of mood and other anxiety disorders (See S1 Table). The summary OR (1.01; 95% CI: 0.81–1.22) indicated that there was no association between SAD and reproduction, and that there was considerable heterogeneity between the studies (I^2^ = 79%). Furthermore, in the longitudinal analysis of the NCS 10-year follow-up data, there was no evidence of an association between SAD at baseline and reproduction at follow-up (OR: 1.16; 95% CI: 0.93–1.44).

### Alcohol Use Disorder (AUD)

We then assessed whether a lifetime history of AUD was associated with reproduction. There was a positive association between lifetime history of AUD and reproduction (Summary OR: 1.09; 95% CI: 1.03–1.15). Similarly, in the longitudinal analysis of the NCS 10-year follow-up, AUD at baseline was positively associated with reproduction at follow-up (OR: 1.46; 95% CI: 1.16–1.85).

### SAD and AUD

**Fig 1:**
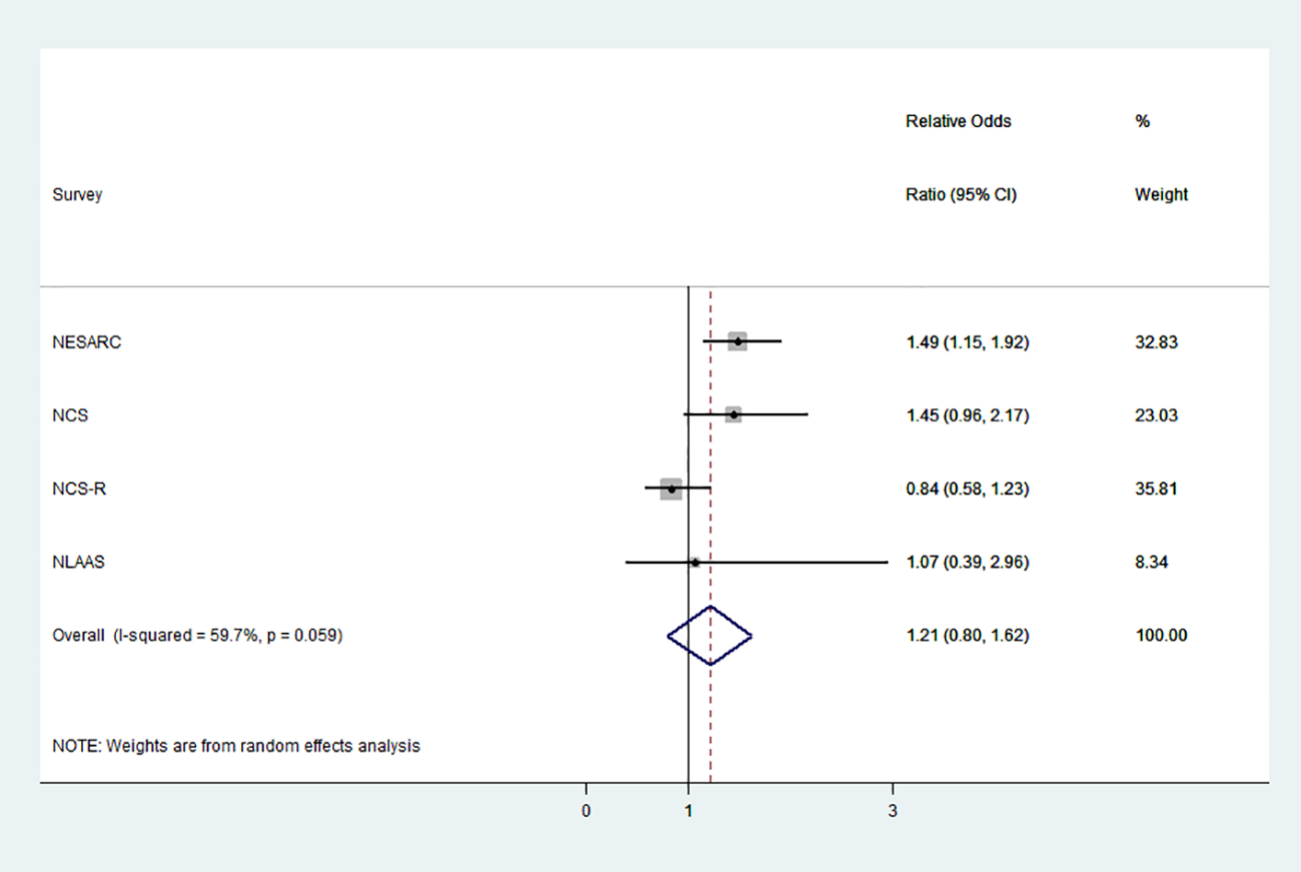
The association between SAD with versus without AUD and reproduction. Relative Odds Ratios (RORs; black dots) and 95% confidence intervals (horizontal black lines) for SAD with versus without AUD and reproduction. The solid vertical line demarcates between negative (left) and positive (right) effects. The gray boxes are proportional to the weights corresponding to the studies. The dotted vertical line and center of the hollow blue diamond represent the overall OR, and the width of the diamond the overall 95% CI.

A second binary logistic regression model was fit to assess the association between a lifetime history of SAD with and without AUD as predictors, and reproduction as outcome (See S2 Table). Fig 1 displays the Relative ORs comparing the association between lifetime history of SAD with versus without AUD on reproduction, and the overall OR estimates obtained using random-effects meta-analysis. There were no differences in reproduction observed between the two groups (summary relative OR: 1.21, 95% CI: 0.80–1.62). Similarly, using the NCS 10-year follow-up data, we did not find evidence of an association between joint SAD and AUD at baseline and reproduction at follow-up (relative OR: 0.96; 95% CI: .58–1.56). See S3 Table for the complete output of the analysis.

## Discussion

We predicted that individuals with a lifetime diagnosis of joint SAD and AUD have a higher probability of reproducing relative to those with a lifetime history of SAD alone. Individual participant meta-analyses were used to combine the estimates obtained from four nationally representative samples and cross-validated against longitudinal data from the NCS. The results of the study do not support the hypothesis. However, there was a large heterogeneity between studies: whereas a lifetime history of SAD with AUD was positively associated with reproduction in the NESARC, the association was negative in the NCS-R, and not distinguishable from the null hypothesis in the NCS and the NLAAS. We do not have any clear indication as to the primary drivers of these differences.

A surprising finding of the meta-analysis is that a diagnosis of SAD alone is not negatively associated with reproduction, given that this was a fundamental premise for conducting the present study. It is also surprising that an overall positive and consistent association between AUD and reproduction was observed, given that AUD is associated with adverse health outcomes [42,43]. Together, these findings suggest that AUD may reflect a trade-off between survival and reproduction, with implications for earlier reproductive timing [44], considering that the costs of excessive alcohol consumption include poor reproductive health [45–47]. In contrast to the AUD findings, which indicated consistency between the individual studies, there was considerable heterogeneity between the individual studies in terms of the association between SAD and reproduction. The high heterogeneity implies that there are evidently other important variables that contribute to this relationship, which can include methodological differences between studies (e.g. measurement), selection biases that affect the composition of the SAD group (e.g. etiology, sub-types) and other factors.

There may also be important effect modifiers that we were unable to assess. For example, trait impulsivity is a strong risk factor for early initiation of drug use in adolescents [48,49], and socially anxious young adults with high trait impulsivity are more likely to initiate alcohol consumption and drink excessively [50], among engaging in other risk-taking behaviors. Personality variables such as impulsivity may underpin increased alcohol use and reproduction. Similarly, findings from network studies suggest that alcohol consumption spreads principally between friends and relatives [51–53], and it may be that the probability of developing AUD, finding a mate and reproducing are all independently contingent on social network features (for instance, connections to social groups where drinking may also tend to occur more frequently). It is also important to note that our data speak only to the question of current fitness, and cannot be used to directly address questions about past utility or phylogeny [54–56].

Limitations of the present study pertain to the type of data used to address the research question. First, the use of cross-sectional data for the meta-analyses precludes the assessment of the associations in a way that incorporates temporal information. We sought to address this limitation by cross-validating the findings of the meta-analysis against 10-year follow-up data from the NCS. Second, the use of dimensional measures of present social anxiety and alcohol use patterns would better capture the relationships between these variables than retrospectively ascertained social anxiety and alcohol use (or lifetime SAD and AUD) that is prone to recall error. Third, the assessment of reproduction is best performed among people who are at or near the end of their reproductive careers. Therefore, the most suitable data for addressing this question would be longitudinal, with extensive follow-up assessments from early adulthood to middle-age. Another limitation pertains to the lack of other proxies for fitness besides the use of the binary reproduction variable. For example, the samples were drawn from a society with wide-ranging access to contraceptives, which may create a disjunction between the deliberate choice not to reproduce and being unfit to do so.

### Conclusions

These findings do not support the notion that SAD is negatively associated with reproduction. In contrast, AUD, which poses fitness costs in terms of survival [42] and reproductive health [45,46], is positively associated with reproduction. Excessive drinking may reflect an evolutionary tradeoff between longevity and reproduction, however this question should be addressed more thoroughly based on the preceding recommendations. Finally, there was no evidence for the hypothesis that individuals with a lifetime history of SAD and AUD reproduce more than individuals with a lifetime history of SAD without AUD.

## Supporting information

S1 Table The association of lifetime SAD and AUD and reproduction in four national samples of the USA population. (DOCX)

S2 Table Association between lifetime SAD with and without AUD and reproduction in four national samples of the USA population. (DOCX)

S3 Table Longitudinal analysis of the relationship between lifetime SAD with and without AUD and reproduction over a 10-year period in Waves 1 and 2 of the National Comorbidity Surveys. (DOCX)
