# Supplementary material for: The association of Social Anxiety Disorder, Alcohol Use Disorder and reproduction: Results from four nationally representative samples of adults in the USA
Source: PLoS One. 2017 Nov 21;12(11):e0188436. doi: 10.1371/journal.pone.0188436 (PMC5697818; doi:10.1371/journal.pone.0188436)
Supplement: S1 Table — (DOCX) [file pone.0188436.s001.docx]

| S1 Table. *The association of lifetime SAD and AUD and reproduction in four national samples of the USA population* | | | | | |
| --- | --- | --- | --- | --- | --- |
| Odds Ratios (and 95% CI) | | | | | |
|  | | NESARC | NCS | NCS-R | NLAAS |
| Age | | 1.08 (1.07-1.08) | 1.16 (1.14-1.19) | 1.08 (1.07-1.09) | 1.11 (1.09-1.12) |
| Sex  Male  Female | | 1 1.55 (1.47-1.65) | 1  1.66 (1.35-2.04) | 1  1.74 (1.57-1.92) | 1  2.04 (1.67-2.50) |
| Education  Bachelor’s degree or higher  Some college  Completed high school  Less than high school | | 1  1.35 (1.25-1.45)  1.69 (1.54-1.86)  2.28 (1.98-2.63) | 1  2.05 (1.50-2.79) 3.12 (2.15-4.53) 2.55 (1.72-3.79) | 1  1.48 (1.23-1.78) 2.14 (1.71-2.68) 2.38 (1.86-3.04) | 1  2.24 (1.80-2.80) 3.50 (2.72-4.49) 5.90 (4.36-7.99) |
| Lifetime Anxiety Disorders^1^  No  Yes | | 1  1.11 (1.00-1.21) | 1  1.39 (1.08-1.78) | 1  1.16 (0.95-1.40) | 1  0.71 (0.47-1.08) |
| Lifetime Mood Disorders^2^  No  Yes | | 1  1.00 (0.93-1.07) | 1  0.93 (0.74-1.17) | 1  0.97 (0.86-1.09) | 1  0.93 (0.72-1.21) |
| Lifetime SAD  No  Yes | | 1 0.81 (0.71-0.92) | 1 1.06 (0.91-1.23) | 1 1.18 (1.00-1.40) | 1  1.09 (0.72-1.66) |
| Lifetime AUD  No  Yes | | 1  1.09 (1.02-1.16) | 1  1.15 (0.96-1.38) | 1  1.07 (0.93-1.24) | 1  0.99 (0.69-1.44) |
|  | *F*(9, 57) = 298.12  VIF: 1.11 | | *F*(9, 34) = 46.22  VIF: 1.11 | *F*(9, 34) = 80.47  VIF: 1.11 | *F*(9, 61) = 40.06  VIF: 1.10 |
| ^1^Panic Disorder, Generalized Anxiety Disorder, and Simple Phobias  ^2^Major depression, Dysthymia, Manic or Hypomanic disorder  VIF: Variance Inflation Factor | | | | | |
