# Supplementary material for: The association of Social Anxiety Disorder, Alcohol Use Disorder and reproduction: Results from four nationally representative samples of adults in the USA
Source: PLoS One. 2017 Nov 21;12(11):e0188436. doi: 10.1371/journal.pone.0188436 (PMC5697818; doi:10.1371/journal.pone.0188436)
Supplement: S2 Table — (DOCX) [file pone.0188436.s002.docx]

| S2 Table. A*ssociation between lifetime SAD with and without AUD and reproduction in four national samples of the USA population* | | | | | |
| --- | --- | --- | --- | --- | --- |
| Odds Ratios (and 95% CI) | | | | | |
|  | | NESARC | NCS | NCS-R | NLAAS |
| Age | | 1.08 (1.07-1.08) | 1.16 (1.14 – 1.19) | 1.08 (1.07-1.09) | 1.11 (1.09-1.12) |
| Sex  Male  Female | | 1 1.53 (1.45-1.62) | 1  1.63 (1.33-2.00) | 1  1.72 (1.55-1.90) | 1  2.04 (1.70-2.47) |
| Education  Bachelor’s degree or higher  Some college  Completed high school  Less than high school | | 1  1.35 (1.25-1.45)  1.69 (1.53-1.86)  2.26 (1.96-2.61) | 1  2.06 (1.52-2.80)  3.14 (2.17-4.55)  2.55 (1.72-3.78) | 1  1.49 (1.24-1.78) 2.15 (1.72-2.70) 2.41 (1.88-3.09) | 1  2.24 (1.80-2.80) 3.50 (2.71-4.51) 5.90 (4.39-7.93) |
| Lifetime Anxiety Disorders^1^  No  Yes | | 1  1.11 (1.02-1.22) | 1  1.40 (1.08-1.80) | 1  1.17 (0.96-1.42) | 1  0.71 (0.47-1.08) |
| Lifetime Mood Disorders^2^  No  Yes | | 1  1.00 (0.94-1.08) | 1  0.95 (0.76-1.19) | 1  0.98 (0.87-1.11) | 1  0.93 (0.72-1.19) |
| Lifetime SAD  No  Without AUD  With AUD | | 1 0.67 (0.56-0.80) 1.00 (0.83-1.19) | 1 0.94 (0.75-1.17) 1.36 (0.97-1.92) | 1 0.89 (0.72-1.10) 0.75 (0.55-1.02) | 1  0.90 (0.50-1.63) 0.96 (0.42-2.21) |
|  | *F*(9, 57) = 299.58 VIF: 1.09 | | *F*(9, 34) = 41.52  VIF: 1.09 | *F*(9, 34) = 87.09 VIF: 1.09 | *F*(9, 61) = 45.60  VIF: 1.08 |
| ^1^Panic Disorder, Generalized Anxiety Disorder, and Simple Phobias  ^2^Major depression, Dysthymia, Manic or Hypomanic disorder  VIF: Variance Inflation Factor | | | | | |
