# Supplementary material for: The association of Social Anxiety Disorder, Alcohol Use Disorder and reproduction: Results from four nationally representative samples of adults in the USA
Source: PLoS One. 2017 Nov 21;12(11):e0188436. doi: 10.1371/journal.pone.0188436 (PMC5697818; doi:10.1371/journal.pone.0188436)
Supplement: S3 Table — (DOCX) [file pone.0188436.s003.docx]

| S3 Table. *Longitudinal analysis of the relationship between lifetime SAD with and without AUD and reproduction over a 10-year period in Waves 1 and 2 of the National Comorbidity Surveys* | |
| --- | --- |
| Odds Ratios (and 95% CI) | |
| Age | 0.84 (0.83-0.86) |
| Sex  Male  Female | 1  0.77 (0.65-0.90) |
| Education  Bachelor’s degree or higher  Some college  Completed high school  Less than high school | 1  0.50 (0.35-0.70) 0.38 (0.25-0.60) 0.22 (0.15-0.33) |
| Lifetime Anxiety Disorders^1^  No  Yes | 1  0.95 (0.69-1.32) |
| Lifetime Mood Disorders^2^  No  Yes | 1  0.98 (0.74-1.31) |
| Lifetime SAD  No  Without AUD  With AUD | 1  1.23 (0.93-1.63)  1.18 (0.79-1.76) |
|  | *F*(9, 34) = 68.73  VIF: 1.09 |
| ^1^Panic Disorder, Generalized Anxiety Disorder, and Simple Phobias  ^2^Major depression, Dysthymia, Manic or Hypomanic disorder  VIF: Variance Inflation Factor | |
